# Supplementary material for: Elastodontic Appliances for the Interception of Malocclusion in Children: A Systematic Narrative Hybrid Review
Source: Children (Basel). 2023 Nov 17;10(11):1821. doi: 10.3390/children10111821 (PMC10670240; doi:10.3390/children10111821)
Supplement: Supplementary file 1 [file children-10-01821-s001.zip › children-2696550-supplementary.pdf]

Supplementary Table S1. Characteristics of the included primary studies
Articles included in the Narrative review

Author/ year	Sample	type of study	Objective
Chen, 2019	1 patient	Case Report	The purpose of this case report is to propose a novel two-phase protocol for extraction instances that may benefit patients as well as doctors. The procedure involves the use of a new elastodontic appliance in the first phase of treatment.
Ciavarella, 2021	40 patients	Case Control	The study's objective was to assess how the elastodontic appliance (EA) affected patients' cephalometric results while they were treated for skeletal Class II/1 malocclusions.
Fichera, 2021	40 patients	Retrospective study	This study evaluated the skeletal and dentoalveolar alterations in a retrospective cohort of children who reported early signs of malocclusion following a year of therapy with elastodontic appliances (EA). A thorough explanation of the evaluated EAs was also provided.
Ierardo, 2017	1 patient	Case Report	The study's objective was to describe the application of elastodontic therapy in a developing patient with second-class malocclusion, lower arch crowding from deciduous to permanent dentition, deep bite, and Dentinogenesis Imperfecta.

Inchingolo, 2022	4 patients	Case series and literature overview	The purpose of this study was to give an overview of elastodontics, including its history, indications, and limitations, as well as to detail four instances that were treated with novel elastomeric devices called AMCOP Bio-Activators.
Inchingolo, 2022	21 patients	Retrospective study	The current study examines the impact on upper airway patency and the efficacy of elastodontic therapy using AMCOP® devices in treating children with hyperdivergent class II malocclusion.
Laganà, 2010	1 patient	Case Report	The purpose of this report is to provide a case study of a patient who had severe deep bite, increased overjet, retrognatic and gummy smiles, class II skeletal and dental malocclusion. This case was resolved with only Occlus-o-guide® during mixed dentition.
Lo Giudice, 2022	36 patients	Prospective study	The current prospective study was designed to assess palatal morphological development during treatment as well as the efficacy of elastodontic appliances (EAs) in treating subjects with Class II sagittal discrepancy in mixed dentition.

Lo Giudice, 2023	39 patients	Retrospective study	The objective of the current study was to assess how the use of elastodontic devices (EAs) treated functional posterior crossbite (FPXB) in terms of changes in palate morphology and dimension.
Marra, 2022	40 patients	Retrospective study	The current study aimed to evaluate the impact of A.M.C.O.P. Bio-activator therapy on the occurrence of atypical swallowing in growing patients, specifically with regard to tongue thrust.
Ortu, 2021	60 patients	Comparative study	The purpose of this study was to confirm the effectiveness of two elastodontic devices, the Occlus-o-Guide (Sweden & Martina) and Equilibrator Series II (Eptamed), in reducing overjet (OJ) and overbite (OB) during treatment.
Patano, 2023	68 patients	Case Control	In this study, the effects of functional elastodontic devices on patients with skeletal Class II malocclusion were compared to an untreated control group in order to examine changes in the dimensions of the hyoid bone and upper airway.
Ronsivalle, 2023	20 patients	Retrospective study	The current study compared the changes in children treated with bimaxillary removable plates supported by class III elastics and elastodontic devices for mild class III malocclusion.

Supplementary Table S2. Articles excluded after full-text evaluation, with reasons (n=107)

Article excluded	Reasons for exclusion
(Assa 1994)	5
(Agostino 2014)	1
(Al-Taai 2023)	5
(Almasoud 2017)	5
(Alqerban 2018)	5
(Alsawaf 2022)	1-2
(Alwadei 2023)	5
(Anderson 2009)	5
(Andrade 2019)	5
(Anhouri 2009)	5
(Antonarakus 2021)	5
(Araujo 2023)	2
(Armi 2021)	5
(Barlow 2009)	5
(Barth 2018)	5
(Bastos 2023)	1
(Batwa 2018)	5
(Baxman 2010)	5
(Bazargani 2014)	5
(Bedoya 2009)	3
(Begum 2014)	5
(Berman 2023)	5
(Bistaffa 2023)	5
(Bonacci 2011)	5
(Borrie 2014)	5
(Bronson 2014)	5
(Bucci 2023)	1-2
(Burhan 2016)	5
(Cai 2014)	5
(Cannavale 2013)	5
(Cao 2016)	5
(Cardoso 2015)	5
(Carli 2023)	5
(Celli 2018)	5
(Changsiripun 2023)	5
(Chen 2019)	1-2
(D'Apuzzo 2019)	5
(Da Silva 2023)	5
(Dash 2023)	5
(De Bito 2018)	5
(Dias 2019)	5

(Einy 2020)	1
(El 2020)	5
(Elnaghy 2022)	5
(Espinosa 2021)	1-2
(Fareen 2017)	5
(Fleming 2017)	5
(Garib 2019)	4
(Garib 2021)	5
(Gili 2021)	2
(Giordano 2019)	5
(Goracci 2017)	5
(Guarnieri 2022)	5
(Guzzo 2014)	4
(Habib 2023)	5
(Haiim 2011)	5
(Hamid 2013)	5
(Hasanin 2021)	5
(Helm 2021)	5
(Hsiao 2016)	5
(Hu 2015)	1
(Isci 2010)	5
(Ismail 2022)	5
(Jaju 2009)	5
(Jamilian 2012)	5
(Janson 2014)	5
(Jolley 2010)	5
(Jung 2018)	5
(Kassir 2011)	5
(Keim 2013)	4
(Kidner 2016)	5
(King 2012)	5
(Kolokitha 2023)	5
(Kouvelis 2018)	1
(Kravitz 2019)	5
(Kumar 2016)	5
(Lakhani 2023)	5
(Le Gall 2011)	5
(Lee 2012)	5
(Lee 2013)	5
(Levin 2012)	5
(Li 2022)	5
(Lindsten 2013)	5
(Liu 2020)	5
(Lopes 2021)	5

(Majorana 2015)	5
(Manni 2011)	5
(Manzoor 2023)	5
(Mazzoleni 2014)	5
(Mew 2012)	5
(Mittal 2017)	5
(Moda 2023)	1
(Mostafiz 2019)	3
(Naoumova 2014)	5
(Ng 2008)	5
(Ngom 2011)	5
(Nota 2021)	5
(Olive 2017)	5
(Paglia 2023)	5
(Park 2012)	5
(Pellegrino 2020)	5
(Perillo 2011)	5
(Piancino 2017)	5
(Prado 2019)	5
(Quinzi 2020)	5
(Redua 2020)	5
(Sajnani 2015)	3

ƚ Reasons for exclusion: 1-Systematic Review article; 2- Meta-analysis; 3-Review or scoping review; 4- Interview/editorial; 5- Topic not compatible with the subject of the study;
